# Transient Aortic Vasospasm Case Report—The “Triple Adrenal” Sign

**DOI:** 10.1155/crra/8910069

**Published:** 2026-04-13

**Authors:** Adam Katz, Alexa DeMaio, Peter Frech, Dell P. Dunn

**Affiliations:** ^1^ Spencer Fox Eccles School of Medicine, University of Utah, Salt Lake City, Utah, USA, utah.edu; ^2^ University of Utah Department of Radiology and Imaging Sciences, University of Utah, Salt Lake City, Utah, USA, utah.edu; ^3^ George E. Wahlen Department of Veterans Affairs Medical Center, Veterans Affairs Salt Lake City Healthcare System, Salt Lake City, Utah, USA

**Keywords:** aorta, artery, case report, vasospasm

## Abstract

Arterial vasospasm is a clinically significant entity that can lead to significant morbidity and, in some cases, even mortality. Although vasospasm most commonly occurs in coronary and cerebral arteries, it can occur in any vessel, including the aorta. This case report describes a favorable clinical outcome following prompt recognition and management and introduces a memorable imaging sign—the “triple adrenal”—to aid identification. To reduce adverse outcomes, radiologists should be aware of aortic vasospasm and the agents and conditions that precipitate it. Given its rarity, aortic vasospasm may be challenging to diagnose. As with any ischemic event, reducing time to reperfusion is critical in preventing irreparable damage.

## 1. Introduction

Arterial vasospasm is mediated by molecules that induce contraction of the smooth muscles within arterial walls [1, 2]. Vasospasm may be caused by sympathetic stimulation, smooth muscle hyperactivity, or stimulant‐associated vasospasm [1] and is often induced by external triggers such as medications, recreational drugs, and autoimmune conditions. Vasospasm may also occur as a physiologic response in conditions such as shock and hypothermia.

Although relatively common in cerebral and coronary arteries [3, 4], vasospasm of the aorta is a rare entity with only a few case reports and an unknown incidence in our review of the medical literature [5]. Here, we report the case of a 44‐year‐old man who, after presenting with abdominal pain, was found to have transient aortic vasospasm.

## 2. Case Presentation

Written informed consent for publication was obtained from the patient. A 44‐year‐old man with a past medical history of hypertension, hyperlipidemia, tobacco use disorder, alcohol use (12 beers per week), and attention‐deficit hyperactivity disorder (ADHD) presented with acute abdominal pain, which started after lifting a 50‐lb object. His pain gradually increased and then acutely worsened, prompting his visit to the emergency department. He also endorsed subjective fevers, chills, nonbloody nonmelanotic diarrhea, and calf claudication. He had no history of prior abdominal surgeries. Pertinent medication included prescribed Adderall (dextroamphetamine and amphetamine) 20 mg BID and filled monthly, which the patient reported just prior to this pain episode (prescription confirmed in medical record on February 3, 2026). His vital signs were normal. His physical exam was notable for abdominal tenderness. 2+ peripheral pulses were palpated. Other than an elevated lactate at 2.4 mmol/L, he had normal labs, including CBC with differential and CMP. A urine toxicology test was not obtained, but the patient was followed monthly by his primary care doctor who intermittently checked urine drug screens, the last one being 5 months prior. The patient never had a positive test for nonprescribed controlled substances.

Standard CT of the abdomen and pelvis (Siemens Somatom Definition AS+, 3‐mm axial slice thickness, 112‐mL Isovue 350 at 2.3 mL/s, portal venous phase) to evaluate the patient′s symptoms showed severe narrowing and concavity of the abdominal aorta and common iliac arteries without evidence of atherosclerosis (Figures 1a, 1b, and 1c). The aorta measured approximately 3‐mm short axis in the affected areas, compared with 20 mm above the diaphragm. A lumbar MRI performed in 2021 was reviewed which showed normal aortic caliber. The CT also showed circumferential narrowing of the celiac axis, superior mesenteric artery, and bilateral renal arteries. There were no CT findings of solid organ infarct or bowel ischemia.

Figure 1(a) Coronal CT (*W*/*L* = 300/40) shows abrupt severe narrowing of the abdominal aorta (white arrow) and renal arteries (blue arrows) beginning at the level of the diaphragmatic hiatus. Note the caliber of the aorta is similar to the left adrenal gland (yellow arrow) with measurements made along the vessel short axis from the inner wall to inner wall of the aorta. The affected portions of the aorta were narrowed to 3 mm as measured 1 cm below the renal arteries compared with 20 mm in the unaffected distal thoracic aorta measured 3 cm above the diaphragm. (b) Axial CT (*W*/*L* = 300/40) of the aortic hiatus shows a concave aortic lumen (white arrow). The narrowed aorta, with its indented appearance, appears similar to the adrenal glands (shown on Figure 1a). (c) Axial CT (*W*/*L* = 300/40) through the pelvis showing flattened and indented bilateral iliac vessels (white arrows).

**Figure:**
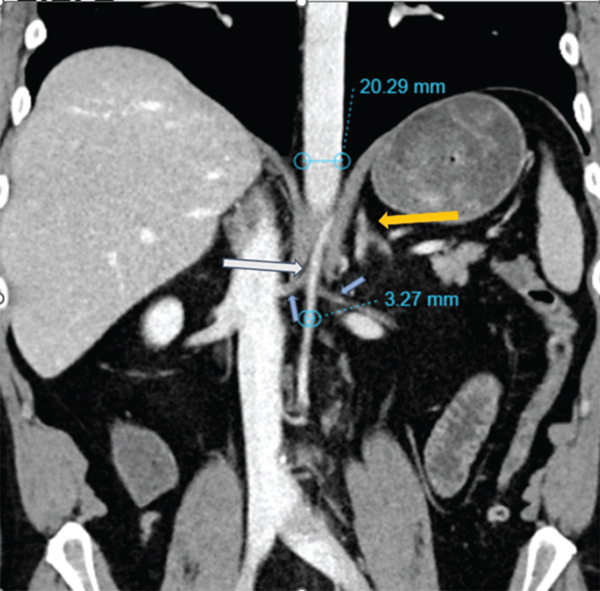
(a)

**Figure:**
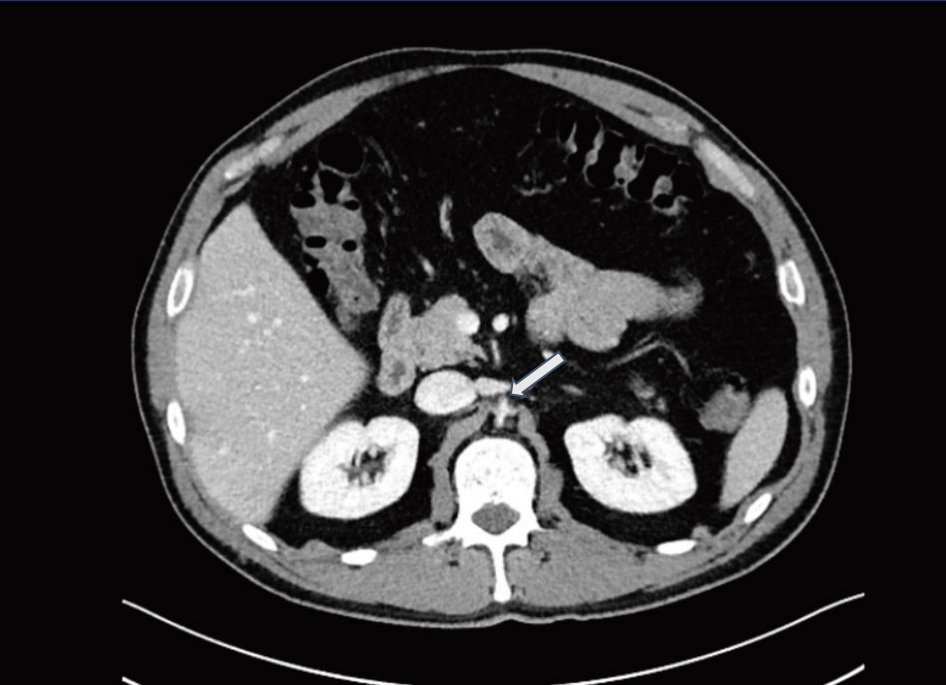
(b)

**Figure:**
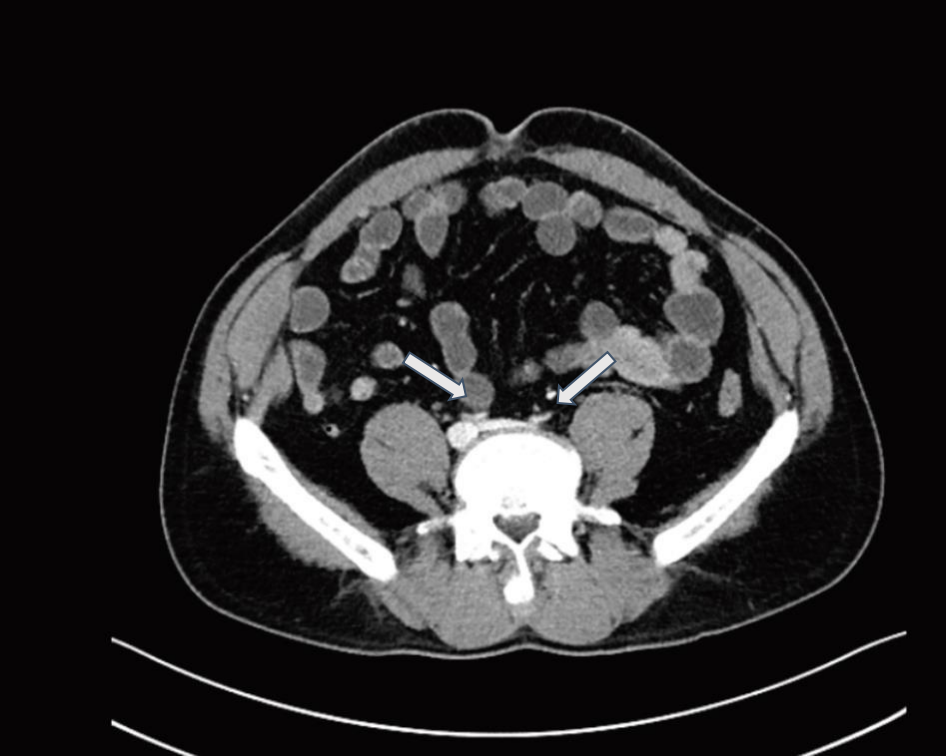
(c)

The initial differential diagnosis included severe vasospasm or a severe vasculitis, perhaps secondary to an autoimmune condition. Since both the imaging findings and the elevated lactate levels suggested hypoperfusion, the patient was admitted to the hospital for fluid resuscitation. Vascular surgery was consulted with concern that the patient might develop acute limb ischemia, acute kidney injury, or mesenteric ischemia. The vascular surgeon determined the patient was clinically stable based on warm extremities and strong 2+ bilateral lower extremity peripheral pulses and opted to admit the patient for supportive care and close monitoring. This monitoring included ESR and CRP levels, aggressive fluid administration, serial lactate levels after 1 L of fluid administration, mental status checks, and pulse checks every 4 h. The patient was not administered a vasodilator due to overall stability with a plan to initiate a vasodilator if peripheral perfusion worsened (such as diminished peripheral pulses or loss of capillary refill), the patient began to show other signs of shock (including oliguria, with set parameters of urine output < 0.5 mL/kg/h for 6 h), or lactate levels continued to rise above the elevated baseline of 2.4 mmol/L.

The following day, the patient′s lactate acid levels had normalized (2.4 → 1.2 mmol/L), CRP and ESR were normal, and an abdominal ultrasound showed a normal caliber aorta without evidence of collapse, narrowing, or aneurysm. The aorta also remained normal in caliber throughout the cardiac cycle and during Valsalva maneuvers. Later, on a CT angiogram performed 48 h after presentation (Siemens Somatom Edge Plus, 2‐mm axial slice thickness, 100‐mL Omnipaque 350 at 4.0 mL/s, early arterial phase), the abdominal aorta and its branch vessels had all returned to normal caliber (19 mm in the previously affected area). The patient was ultimately discharged with a diagnosis of aortic vasospasm and a recommendation for a single follow‐up CTA in 6 months to exclude asymptomatic recurrence. The patient agreed with this treatment plan. For a summarized timeline of this case presentation and episode of care, please view Table 1.

**Table 1:** Timeline of patient′s presentation and clinical course.

Arrival to emergency department	Patient reports acute abdominal pain following lifting a 50‐lb object. Also reports taking Adderall shortly before the onset of pain. Found to have lactate of a 2.4 mmol/L. Standard CT of the abdomen and pelvis shows severe narrowing and concavity of the abdominal aorta and common iliac arteries.
Admission to hospital	Fluid resuscitation initiated. Vascular surgery consulted and determined the patient was stable with strong 2+ bilateral lower extremity peripheral pulses, and opted to admit the patient for supportive care and close monitoring.
24 h later	Patient′s lactate level dropped to 1.2 mmol/L. Abdominal ultrasound showed a normal caliber aorta. Vitals remained stable.
48 h later	CT angiogram shows the abdominal aorta and its branch vessels had all returned to normal caliber. Patient is discharged with plans for follow‐up CTA in 6 months.

## 3. Discussion

This case demonstrates hemodynamically significant transient aortic vasospasm, summarized in Table 2. At the time of clinical presentation, early differentials included drug‐induced vasospasm, hypothermia, severe vasculitis, or shock. However, the patient′s normal vital signs, benign physical exam without findings to suggest shock, and normal inflammatory markers (ESR and CRP levels), combined with absent intimal flap, no double lumen, and minimal wall thickening on imaging (consistent with dissection and vasculitis, respectively), eliminated many differentials. The resolution of symptoms without intervention ruled out all but one possibility—vasospasm.

**Table 2:** Summary of the case presentation, including management and outcome.

Presentation	—Acute abdominal pain after lifting 50‐lb object —Elevated lactate (2.4) upon admission —Vital signs are normal
Suspected trigger	Adderall
Vessel involvement	CT findings of concentric narrowing of the abdominal aorta and common iliac arteries
Management	—Admitted to hospital for fluid resuscitation, vascular surgery consult —Underwent 24‐h monitoring, workup included ESR and CRP levels, aggressive fluid administration, repeat lactate levels after 1 L fluid administration, mental status checks, and pulse checks every 4 h
Outcome	—Abdominal ultrasound the next day showed a normal caliber aorta without evidence of collapse, narrowing, or aneurysm —Patient remained stable, lactate resolved —Discharged with recommendation for follow‐up CTA in 6 months

In the absence of evidence for illicit drug use, this aortic spasm was most likely precipitated by the stimulant Adderall; however, without toxicology testing, contribution from other illicit drugs such as cocaine cannot be ruled out and the association in this case is inferential. Amphetamines and derivatives are known to cause vasospasm, but most commonly in the extremities and when associated with underlying rheumatologic conditions [6]. Although arterial vasospasm is a well‐known clinical entity and aortic vasospasm has been reported [7, 8], our review of the literature finds only two other case reports of vasospasm causing nearly complete collapse of the aorta [5, 9]. One patient presented with acute intestinal ischemia [5] with an unknown etiology of his vasospasm, whereas another patient presented similarly to ours [9] with the prescribed medication ergotamine as the favored trigger for aortic vasospasm.

**Figure 2:**
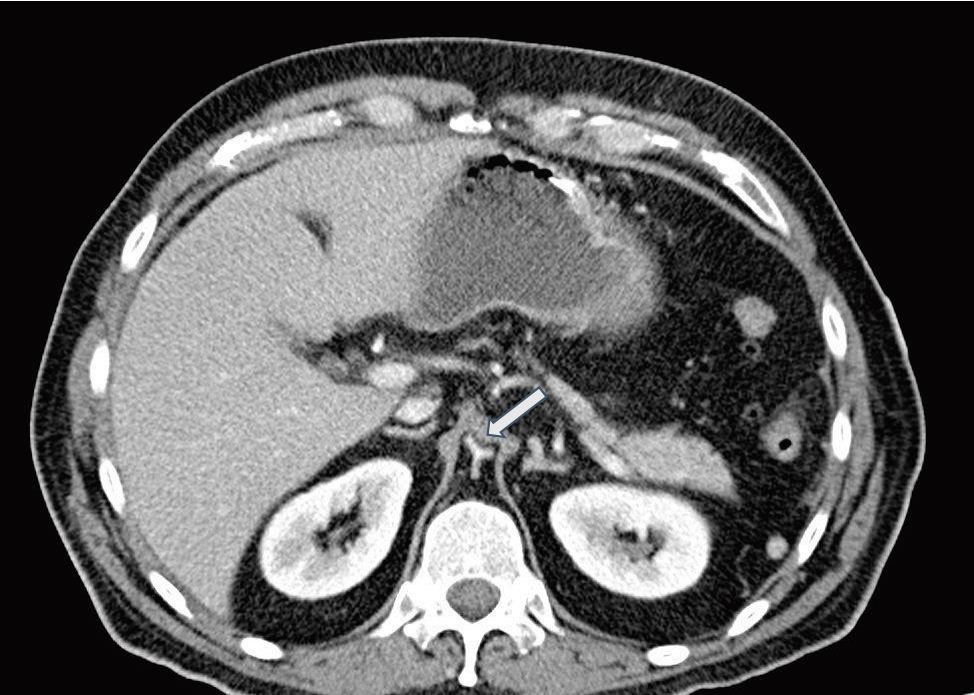
Companion case of aortic vasospasm. This case depicts an excellent “triple adrenal” sign (white arrow). Image reprinted with permission from Pérez‐Alonso et al. [5]

Given the rarity of this condition, clinicians and radiologists may find its diagnosis difficult. Unlike other vascular pathologies, when spasm is present, the abdominal aorta can have a caliber and appearance similar to the adrenal glands, appearing almost as a fleur‐de‐lis pattern, resulting in the novel “triple adrenal sign” as shown by our case in Figure 1b as well as a similar case in the literature as shown in Figure 2. Of note, this sign, although manifesting in other abdominal aortic vasospasm case reports, should not be considered a diagnostic image finding but can still be a strong heuristic to support the radiologist when considering this rare diagnosis.

Aortic vasospasm can be considered a subset of nonocclusive mesenteric ischemia. Autoimmune conditions or shock may be the cause of ischemia, but careful medication reconciliation and open communication about recreational drug use are key to identifying potential causative agents. In this case, the patient had taken Adderall; however, patients using nonprescribed stimulants (i.e., amphetamines and cocaine) may not offer this up as part of a routine history. From this case and the few examples in the literature, it appears that medication‐induced aortic vasospasm responds well to conservative management with aggressive supportive care, but identifying inciting factors is important to prevent future repeat events. Vasodilators can be administered when there is evidence of significant end‐organ ischemia.

This case substantiates that positive patient outcomes can be achieved in patients with vasospasm through supportive management and close serial evaluation in stable patients without clinical, laboratory, or radiologic findings to suggest severe disease. For example, in one of the few other cases of aortic vasospasm in the literature, the patient had marked signs of hypoperfusion including elevated inflammatory markers, white blood cells, and dilated loops of small bowel, which were absent in our case [9]. At the clinicians′ discretion, or in more severe presentations, patients may require more active management with vasodilator therapy. This may be considered if patients are refractory to supportive care or display signs of worsening/increasing hypoperfusion or ischemia. Thresholds for intervention include rising lactate (trend more important than specific levels) and ongoing signs of peripheral hypoperfusion such as diminished peripheral pulses, loss of lower extremity capillary refill, anuria or rising creatinine. Though in this case, our patient′s condition resolved without any observed permanent ischemic changes, repeat episodes of the inciting factor could lead to permanent ischemic damage, as can be seen in cocaine‐induced coronary vasospasm leading to myocardial infarction [2, 3]. Identifying and avoiding the causative agent is important even after symptom resolution. Long term, the authors recommend continued surveillance with single CTA at 6 months to exclude asymptomatic recurrence. If recurrent or more symptomatic vasospasm occurs, amphetamine‐based stimulants should be discontinued. For radiation‐sensitive patients (including pregnant patients), abdominal ultrasound could be a viable surveillance method.

This case also highlights the importance of cooperation between radiologists and clinicians in identifying and managing cases of aortic vasospasm. In our case, coordination with vascular surgery led to quick diagnosis and cessation of the inciting agent and good supportive care, resulting in full recovery before irreversible damage occurred. Though aortic vasospasm is rare, its rapid recognition and treatment are important to avoid significant morbidity and possible mortality. The “triple adrenal” sign is a simple mnemonic that could help radiologists remember to include vasospasm in their differential considerations when encountering an abnormally small or collapsed aorta.

## Funding

No funding was received for this manuscript.

## Consent

All the patients allowed personal data processing, and informed consent was obtained from all individual participants included in the study.

## Conflicts of Interest

The authors declare no conflict of interest.

## Data Availability

The supporting data included within this manuscript cited as Figure 2 is available online as open access DOI: 10.1016/j.ciresp.2015.05.011. Complete CT scans from initial presentation and subsequent CTA can be viewed at https://www.pacsbin.com/c/-JL9B94kei
